# Kinetics of fluoride after brushing with the no-rinse method

**DOI:** 10.1186/s12903-024-04807-4

**Published:** 2024-09-08

**Authors:** Tipparat Parakaw, Sirada Srihirun, Pornpen Dararat, Nisarat Ruangsawasdi

**Affiliations:** Department of Pharmacology, Faculty of Dentistry, Mahidol University, Bangkok, Thailand

**Keywords:** No-rinse, Kinetics, Fluoride, Saliva, Plasma, Urine, Adults

## Abstract

**Background:**

Fluoride plays a vital role in preventing dental caries, with its addition to oral care products significantly promoting oral hygiene. A no-rinse brushing method aims to increase fluoride retention in the oral cavity, as rinsing with water decreases fluoride levels in saliva, which could affect remineralization. While the no-rinse brushing method holds promise for improving fluoride retention in the oral cavity, critical inquiries persist regarding its safety. This study investigated the kinetics of oral fluoride and potential risks to fully assess its effectiveness and implications for oral health.

**Methods:**

Ten healthy adults participated in a crossover study comparing the no-rinse with the rinse method. All subjects followed American Dental Association (ADA) brushing guidelines. Levels of fluoride in saliva (supernatant and sediment) and urine were measured over time, and plasma fluoride was measured one hour after brushing. Pharmacokinetic parameters were also calculated from the data.

**Results:**

Participants using the no-rinse method had higher fluoride levels in supernatant immediately and up to 30 min post-brushing compared to the rinse method. Fluoride levels in sediment were higher only immediately after brushing. The total fluoride concentration in saliva remained elevated for up to 5 min with the no-rinse method. Systemic fluoride absorption showed no significant difference between the two methods based on blood and urine analysis.

**Conclusion:**

This research indicates that the no-rinse method can enhance fluoride retention in the oral cavity for up to 30 min after a single brushing. In addition, our findings suggest that this method does not significantly influence systemic fluoride levels or toxicity.

**Registry:**

Thai Clinical Trials Registry, TCTR (http://thaiclinicaltrials.org). Clinical trial registration number: TCTR20231104001 (4/11/2023).

## Introduction

Fluoride is widely recognized as a key in preventing dental caries and maintaining oral health. Toothpaste containing fluoride at 1,000 to 1,500 ppm has effectively prevented tooth decay [1]. Fluoride in the oral cavity can be absorbed by tooth enamel, forming fluorapatite, which strengthens enamel and reduces its susceptibility to acid erosion [2]. This process, known as remineralization, is crucial for preventing dental caries.

Fluoride is typically ingested and absorbed in the gastrointestinal tract with an absorption half-life of 30 min and peak levels in plasma at approximately 1 h [3–6]. Once absorbed, fluoride primarily distributes in plasma and accumulates in mineralized tissues, particularly bones. It can be slowly released from deposit sites when plasma fluoride levels decrease. While fluoride metabolism in the liver is minimal, renal excretion is the primary elimination route, with excretion rates associated with urine pH, glomerular filtration rate (GFR), plasma fluoride levels, and urine flow rate.

The Australian Dental Association has recommended teeth brushing with a no-rinse method twice daily to retain higher fluoride concentration in the oral cavity for extended periods [7]. Public Health England, the Department of Health and Social Care, NHS England, and NHS Improvement also advise spitting out excess toothpaste rather than rinsing as a guidance to promote better oral health [8]. A prior study revealed a correlation between water rinsing, saliva fluoride concentration, and dental caries with individuals who rinsed less having higher salivary fluoride levels and lower incidences of dental caries [9]. Another study found that individuals who often rinsed with water after brushing exhibited a higher incidence of caries than those who did not rinse or only occasionally rinsed during the 3-year observation period [10].

Additionally, researchers have demonstrated that water rinsing decreases the fluoride availability in saliva by 2.5 times [11], and brushing with fluoride toothpaste without rinsing significantly increases fluoride concentration in saliva up to 15 min post-brushing [12]. These findings highlight the importance of the no-rinse method in increasing salivary fluoride levels and prolonging remineralization in the oral cavity. However, the evidence supporting the recommendation of the no-rinse method remains limited, primarily focusing on the impact of fluoride quantity on salivary concentration rather than the effects and safety of this post-rinsing method.

Despite the potential benefits of the no-rinse method, concerns exist regarding fluoride toxicity and accumulation, especially considering the prolonged contact time with fluoride-containing toothpaste. Children and adolescents are particularly sensitive to fluoride toxicity, but adults can also experience symptoms such as skeletal fluorosis and other systemic effects [13, 14]. Long-term fluoride ingestion reported symptoms such as abdominal pain, vomiting, and nausea in 70% of subjects [15]. Abnormalities of histological samples collected from the gastrointestinal tract were also observed. Therefore, it is crucial to consider the potential impact of high fluoride concentration when using the no-rinse method.

Nowadays, concerns about potential fluoride toxicity due to accumulation when people don’t rinse after brushing have emerged. This recent study aimed to assess the kinetics of fluoride retention within the oral cavity, its absorption into the systemic circulation, and its urinary excretion after brushing with the no-rinse method. This research aims to contribute to evidence-based guidelines for the clinical application of fluoride to ensure its effective delivery while prioritizing safety.

## Materials and methods

### Subjects

This study is a crossover design. The study was approved by the Committee on Human Rights Related to Human Experimentation, Mahidol University, Thailand (COA. No. MU-DT/PY-IRB 2023/063.2609). The trial protocol was registered with the Thai Clinical Trials Registry (TCTR) (TCTR20231104001). Sample size calculations were performed using raw data from a prior study [16] with the G*Power program (version 3.1.9.4). The type I error was set at 0.05 with 95% power. From the power analysis, subjects of at least 9 participants were required to participate in the study. We decided to include 10 participants in this study by considering a 10% dropout rate. The participants who reached the following criteria, age between 20 and 35, resting salivary flow rate above 0.3 ml/min, having healthy teeth and gums, no history of liver and kidney disease, and did not take medications that might have affected their salivary flow rate were included in this study. Those who were unable to collect urine every 2 h were excluded. Each participant was conducted both no-rinse and rinse post brushing with at least 7 days of washing period between the rinsing techniques. The washing period refers to the interval between the experiments of the two different rinsing methods, allowing for a clear distinction in the effects of each technique. We selected a 7-day washout period in accordance with guidance from the US FDA on studying bioavailability and bioequivalence that an adequate washout period for cross-over study should be approximately ten times the elimination half-life of the drug [17]. During the washout period, we provided each participant with instructions on maintaining their oral hygiene. Participants were advised to brush their teeth using the Modified Bass technique, as recommended by the ADA, and to avoid any medications that could affect the salivary flow rate throughout the study. To ensure compliance, we contacted participants on day 3 or 4 of the washout period for a follow-up check.

### Brushing and rinsing procedure

**Fig. 1:**
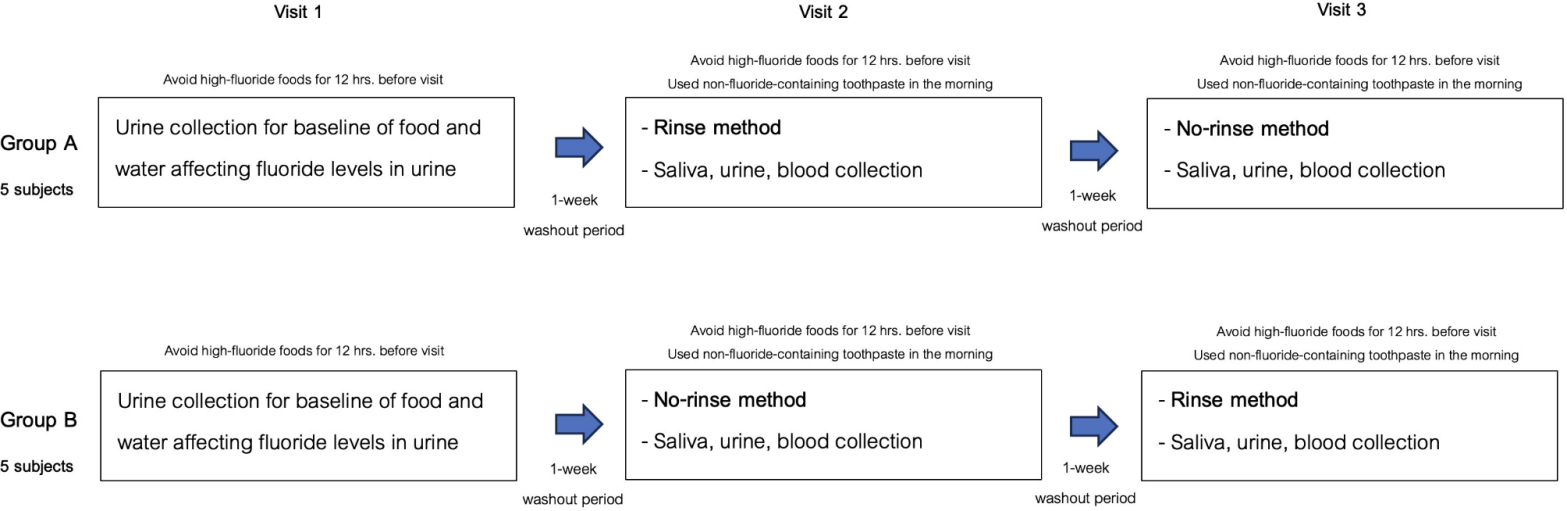
Group allocation and processes from the first visit to the third visit for group **A** and group **B**

To participate in the study, all participants needed to avoid high-fluoride foods for 12 h before attending each experimental visit. During the first visit, urine samples were collected to measure baseline fluoride levels, ensuring the controlled intake of low-fluoride foods didn’t affect urinary fluoride. Participants were then randomly assigned to two groups (group A underwent the rinse method on the second visit and switched to the no-rinse method after a 7-day washout period for the third visit, while Group B started with the no-rinse method on the second visit, had a 7-day washout period, and then switched to the rinse method in the third visit) (Fig. 1). On the days of the second and third visits, fluoride-containing toothpaste was not used in the morning. Participants were instructed to consume only the food (breakfast, lunch) and drink (low fluoride-containing water) provided during the experimental procedure. The experiments were started at least 2 h after breakfast. Each group used either no-rinse method or rinsed with 10 ml of deionized water for 10 s, applying 1 g of pre-weighed toothpaste of the no-rinse toothpaste containing 1500 ppm fluoride (Dentiste Anticavity Max Fluoride Toothpaste, Bangkok, Thailand). Our staff instructed them to follow the American Dental Association (ADA) recommended brushing method, briefly using a modified bass technique with 1 g of pre-weighed toothpaste for 2 min, covering all tooth surfaces.

### Samples collection

Our staff instructed participants to collect unstimulated saliva in a 50 ml sterile tube for 2 min before and immediately, 5, 10, 15, 30, 60, and 90 min after brushing. Urine was collected before, and intervals of 30–60 min, 1–2 h, 2–4 h, and 4–6 h after brushing. Total urine was collected in a 600 ml container at each collection time point to calculate cumulative fluoride excretion. Additionally, 5 ml of blood was collected in a heparinized tube by a registered nurse at 1 h after using the no-rinse method to analyze plasma fluoride. The salivary flow rate of each individual was also determined before brushing by collecting unstimulated saliva in a 50 ml sterile tube for 2 min. The salivary flow rate was measured by calculating the total volume of saliva collected at baseline before brushing with no-rinse formula toothpaste divided by the time of collection.

### Fluoride measurement

**Fig. 2:**
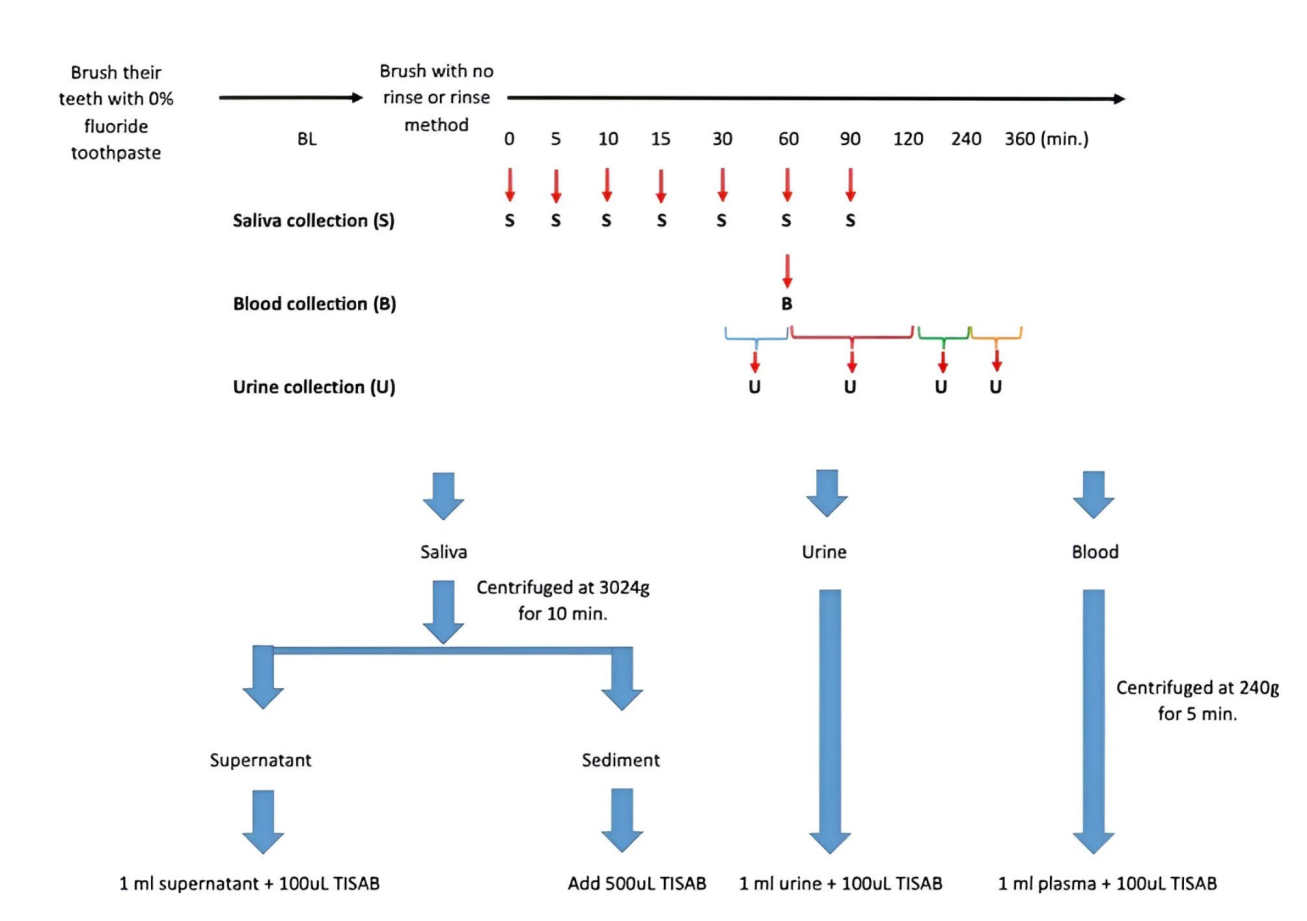
Timeline for sample collection and the process of sample preparation for fluoride measurement

All samples including saliva, blood, and urine were processed differently before evaluating fluoride concentration. Saliva was centrifuged at 3024 g for 10 min to separate supernatant and sediment. The blood sample underwent centrifugation at 240 g for 5 min to obtain plasma. Urine sample volumes were recorded before fluoride measurement. All samples were kept at -20 °C until fluoride analysis using a calibrated ion-specific sensitive electrode (Orion™ Model 9609BNWP, Thermo Fisher Scientific Cambridgeshire, UK). The flow charts below show the complete experimental protocol (Fig. 2).

### Pharmacokinetic of fluoride

The area under the curve (AUC) of total fluoride levels in saliva collected from the no-rinse and rinse groups was calculated from the graph illustrating fluoride levels over time using Prism^®^ version 9 (Prism Software Inc., San Diego, CA, USA). This was done to assess fluoride bioavailability in the oral cavity.

We measured the total urine volume at each collection point to determine the fluoride levels in urine samples. The fluoride quantity was calculated using the following equation.

$$\text{Amount}\:\text{of}\:\text{fluoride} = \text{Fluoride}\:\text{concentration}\:$$

$$\times\:\text{Volume}\:\text{of}\:\text{urine}\:\text{collected}\:\text{at}\:\text{each}\:\text{time}\:\text{point}$$

The cumulative fluoride amount was calculated and plotted against the time of urine collection. Additionally, the excretion rate (fluoride amount/ time) was computed and plotted on a semi-log scale against the collection time. The slope of this graph yielded the elimination rate constant (k_e_).

The plasma fluoride concentration at a single time point was utilized to calculate renal clearance using the following equation.

$$\text{Renal}\:\text{clearance}\:\text{(ml/min)} =$$

$$\:\frac{\text{U}\text{r}\text{i}\text{n}\text{a}\text{r}\text{y}\:\text{f}\text{l}\text{u}\text{o}\text{r}\text{i}\text{d}\text{e}\:\text{c}\text{o}\text{n}\text{c}\text{e}\text{n}\text{t}\text{r}\text{a}\text{t}\text{i}\text{o}\text{n}\:\text{x}\:\text{U}\text{r}\text{i}\text{n}\text{e}\:\text{f}\text{l}\text{o}\text{w}\:\text{r}\text{a}\text{t}\text{e}}{\text{P}\text{l}\text{a}\text{s}\text{m}\text{a}\:\text{f}\text{l}\text{u}\text{o}\text{r}\text{i}\text{d}\text{e}\:\text{c}\text{o}\text{n}\text{c}\text{e}\text{n}\text{t}\text{r}\text{a}\text{t}\text{i}\text{o}\text{n}}$$

Additionally, the volume of distribution (V_d_ ) and half-life (t_1/2_) were calculated using the following equation.

$$\text{Renal}\:\text{clearance} = \:\text{K}_{e} \times \text{V}_{d}$$

$$\text{T}_{1/2} = 0.693 / \text{K}_{e}$$

### Statistical analysis

Statistical analysis was done using IBM SPSS Statistics version 28.0 (IBM Corporation, Armonk, NY) and Prism^®^ version 9 (Prism Software Inc., San Diego, CA, USA). Data are represented as mean ± standard error of the mean (SEM). Histogram and Shapiro-Wilk Test were used for the normality test. A Generalized Estimating Equation (GEE) was used for salivary and urine fluoride analysis. A Paired t-test was used for plasma fluoride analysis, the AUC of fluoride in whole saliva, and the comparison of fluoride in supernatant and sediment after tooth brushing.

## Results

### Demographic profile of study subjects

The study included 4 males (40%) and 6 females (60%). The mean age, height, weight, and body mass index (BMI) of the study population were 22 ± 1.16 years, 166 ± 9.26 cm, 60.5 ± 17.91 kg, and 21.91 ± 4.48 kg/m^2^, respectively. The salivary flow rate of all subjects is in the normal range (average salivary flow rate is 0.58 ± 0.2 ml/min). All characteristics of the study subjects and the mean salivary flow rate are shown in Table 1.

**Table 1:** The demographic profile of the subjects

Characteristics	Mean ± SD
Age (year)	22 ± 1.16
Sex	4 (Male), 6 (Female)
Height (cm)	166 ± 9.2
Weight (kg)	60.5 ± 17.91
BMI (kg/m^2^)	21.91 ± 4.48
Salivary flow rate (ml/ min)	0.58 ± 0.2

### Fluoride concentration in the oral cavity

**Fig. 3:**
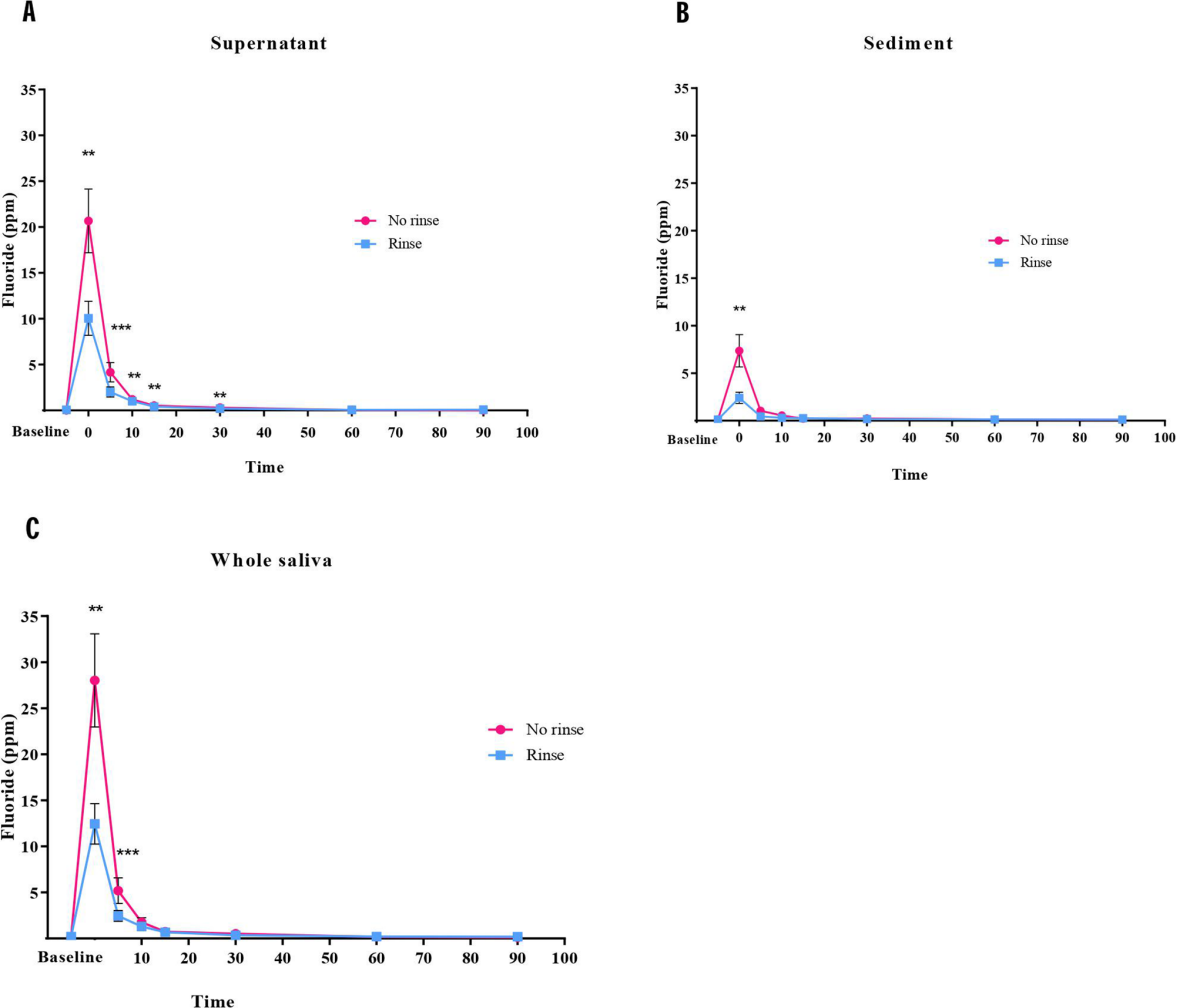
The salivary fluoride concentration of various time points after tooth brushing with no-rinse and rinse method. Fluoride concentrations in saliva were determined in supernatant (**A**), sediment (**B**), and whole saliva (**C**). Data are mean ± SEM (*n* = 10). Statistical significance was determined using a Generalized Estimating Equation (ESS) represented by **P < 0.01, ***P < 0.001

**Fig. 4:**
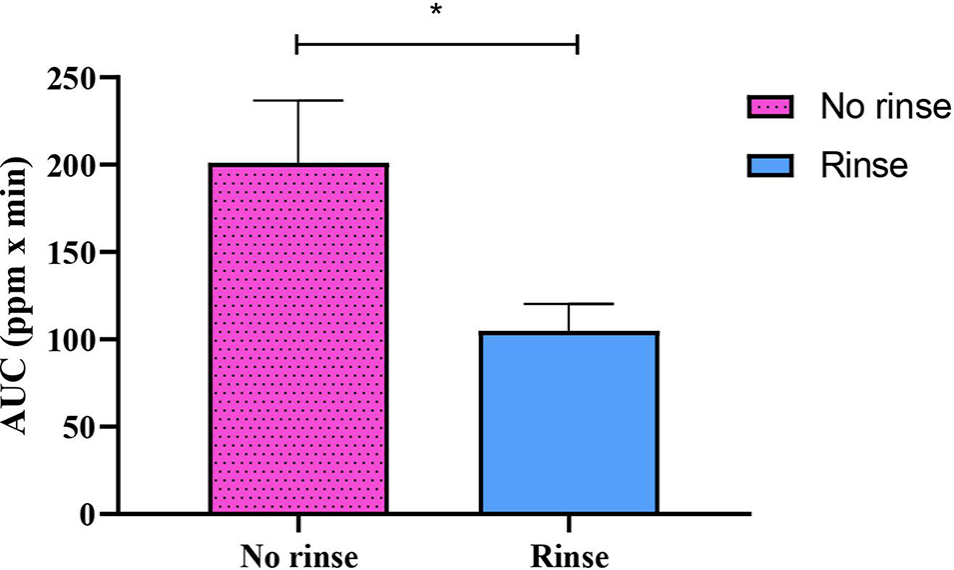
AUC of fluoride in whole saliva after tooth brushing with no-rinse and rinse method. Data are mean ± SEM (*n* = 10). Statistical significance was determined using a Paired t-test represented by *P < 0.05

**Fig. 5:**
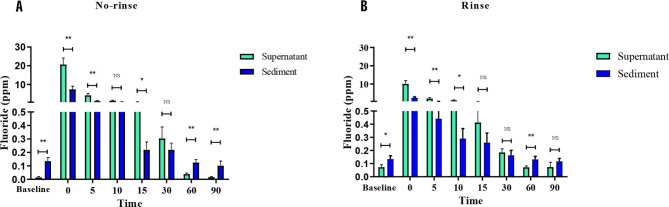
The fluoride concentrations in supernatant and sediment at various time points after tooth brushing with no-rinse (**A**) and rinse (**B**) method. Data are mean ± SEM (*n* = 10). Statistical significance was determined using a Paired t-test represented by *P < 0.05, **P < 0.01

Fluoride concentrations were determined in two compartments of saliva, including supernatant and sediment. Fluoride concentrations in supernatant after brushing following the no-rinse method showed significantly higher than those in supernatant from the rinse method immediately (*p* = 0.02), and 5 min (*p* < 0.001), 10 min (*p* = 0.002), 15 min (*p* = 0.002) and 30 min (*p* = 0.006), respectively after brushing (Fig. 3A). In the sediment compartment, fluoride concentrations in the no-rinse group displayed a significantly higher level than those in the rinse group at only the immediate time point after brushing (*p* = 0.007) (Fig. 3B). When calculated for fluoride in the whole saliva, the no-rinse method exhibited significantly higher levels than those in the rinse group immediately after brushing (*p* = 0.03), and this difference remained statistically significant at 5 min (*p* = 0.01) (Fig. 3C). In addition, the AUC calculated from fluoride in saliva was more critical in the no-rinse than in the rinse technique (*p* = 0.0185) (Fig. 4). Furthermore, we observed that after 60 min following toothbrushing, the sediment had a higher fluoride content than the supernatant in both methods. This pattern of fluoride distribution between the 2 compartments of saliva was also observed at the baseline (Fig. 5).

### Fluoride concentration in plasma

**Fig. 6:**
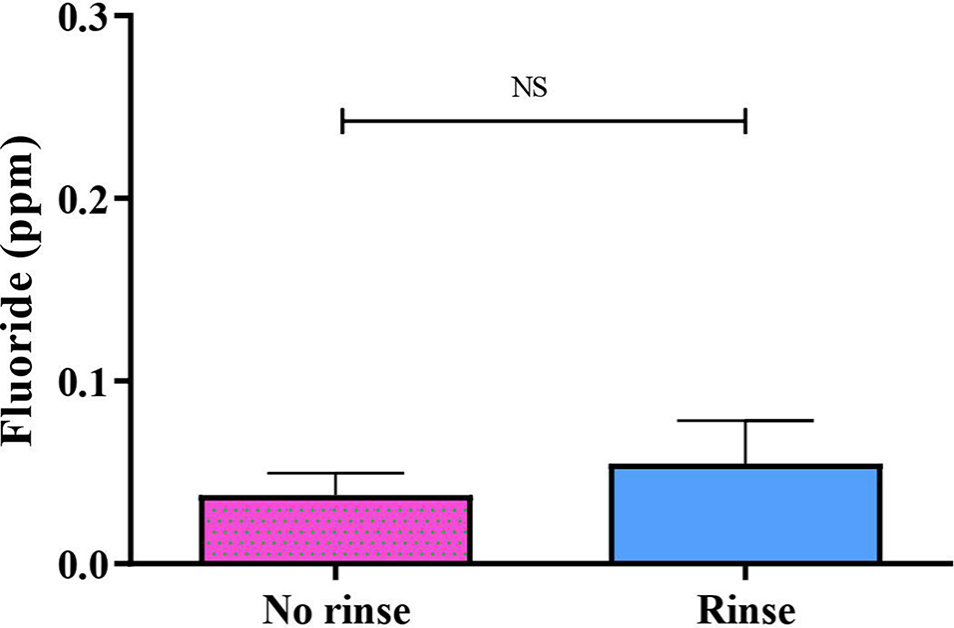
Fluoride concentration in plasma between no rinse and rinse methods at 1 h after brushing. Data are mean ± SEM. *N* = 10 in both groups. Statistical significance was determined using a Paired t-test

Analysis revealed that the no-rinse method resulted in a systemic circulation fluoride concentration at 1 h after brushing of 0.0260 ± 0.12 ppm, while the rinse method exhibited a similar concentration of 0.0234 ± 0.09 ppm (Fig. 6). The statistical analysis revealed no significant disparity between 2 groups (p-value = 0.52).

### Renal fluoride excretion

**Fig. 7:**
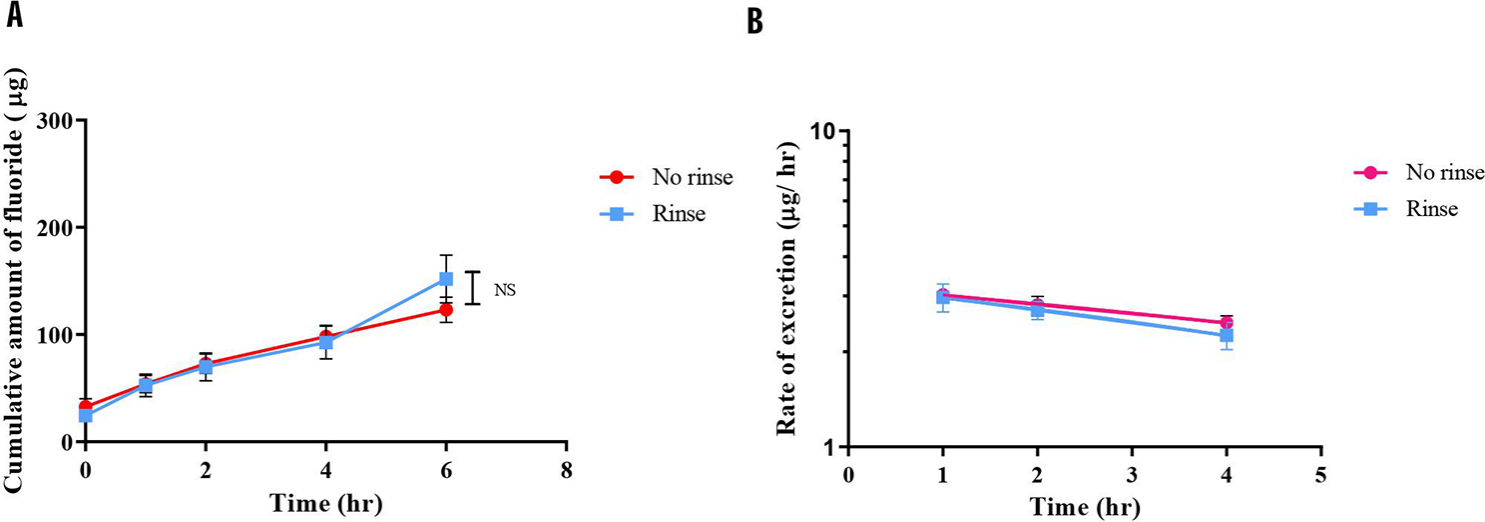
The cumulative amount of fluoride and rate of fluoride excretion of no-rinse and rinse method after brushing. Data are shown as mean ± SEM. *N* = 10 in both groups. Statistical significance was determined using a Generalized Estimating Equation (ESS) for the cumulative amount of fluoride (**A**). The slopes were calculated to obtain the k_e_ for fluoride in both experimental groups (**B**)

The cumulative amount of fluoride was calculated and plotted against the time of urine collection (Fig. 7A). At all time points, no statistically significant differences were observed between the groups (p-value > 0.05). Furthermore, the rate of excretion (amount of fluoride/time) was computed and plotted on a semi-log scale against the time of collection. The slope of this graph provided the elimination rate constant (k_e_), which was 0.19 h^− 1^ for the no-rinse method and 0.23 h^− 1^ (Fig. 7B) for the rinse method.

### Pharmacokinetic of fluoride

From the K_e_ value obtained from the semi-logarithmic graph, we determined the half-life of fluoride after brushing using the no-rinse method to be approximately 3 h and 38 min. This value closely aligns with the half-life calculated from the rinse method, which was approximately 3 h. The renal clearance, estimated from urinary fluoride concentration, urine flow rate, and plasma concentration at 1 h after brushing in the no-rinse group, was 14.36 ml/ min. In contrast, the rinse method yielded an 11.85 ml/ min renal clearance. Furthermore, the volume of distribution (V_d_) estimated from both methods was similar. Specifically, it was determined to be 4.53 L with the no-rinse method and 3.09 L with the rinse method.

## Discussion

This study investigated the kinetics of fluoride in healthy adults following the use of 1500 ppm sodium fluoride toothpaste, specifically employing the no-rinse method after brushing. The research aligned with the recommendations of several dental associations, including the Australian Dental Association, British Dental Association, Nation Health Service (NHS.UK), and Canadian Dental Association, all of which advise against rinsing after brushing. However, there is currently limited data available regarding its efficacy and safety. To bridge this gap, this study compared the no-rinse and rinse methods to assess their impact on fluoride concentrations in supernatant, sediment, and whole saliva. Blood and urine samples were collected at various time points to comprehensively evaluate fluoride absorption to the systemic circulation and excretion. Our findings showed that the no-rinse method resulted in higher levels of fluoride in saliva than the rinse method, particularly immediately after brushing and up to 5 min afterward. Examining saliva components, we found that fluoride concentration peaked in the supernatant immediately after brushing and gradually moved to the sediment. Despite higher salivary fluoride, fluoride levels in plasma and urine are below those observed in patients with fluorosis, suggesting the safety of the no-rinse method after a single brushing.

The no-rinse method consistently resulted in higher fluoride concentrations in both the supernatant and sediment immediately after brushing, and in the supernatant at subsequent time points, compared to the rinse method. A prior study indicated that no-rinsing after brushing with sodium fluoride resulted in higher salivary fluoride levels than the rinse method until 1 min [16]. In contrast, our study showed that using the no-rinse method with sodium fluoride toothpaste sustained greater fluoride levels in the whole saliva for up to 5 min, longer than previously reported. The duration of fluoride exposure in the oral cavity directly influences its effectiveness in promoting remineralization of enamel or dentin. Longer exposure allows fluoride to exert its protective and strengthening effects, contributing to oral health and reducing the risk of tooth decay [18, 19]. If the no-rinse method consistently extended the level of fluoride over 5 min over a period of time, it might show significant remineralization and reduce the incidence of caries. Furthermore, we found that fluoride levels in the supernatant after using the no-rinse method reached the concentrations required to inhibit 50% demineralization (0.3 to 0.4 ppm) [20] for up to 30 min, whereas the rinse method maintained these fluoride levels for up to 15 min after brushing. Our findings align with previous studies, which demonstrated that the no-rinse method maintained salivary fluoride retention in the oral cavity better than the rinse method [21]. Additionally, increased rinsing water decreases fluoride retention in the oral cavity, leading to lower salivary fluoride levels and potentially higher dental caries incidence [9, 12]. Thus, minimizing rinsing after brushing may enhance fluoride efficacy.

This study separated whole saliva into supernatant and sediment components to examine how the rinsing methods (no-rinse Vs. rinse) impact fluoride’s kinetics in both saliva compartments. This approach is crucial as these compartments may influence the bioavailability of fluoride differently within the oral cavity. The cell-free supernatant saliva represents a complex secretion originating from the salivary glands. At the same time, the sediment comprises the bulk of the human oral microbiome, cellular constituents, proteins, and food particles [22]. At baseline (before brushing), fluoride accumulated more in the sediment. Immediately after brushing, fluoride was initially distributed in the supernatant in both methods. However, fluoride levels became more prominent in the sediment after 60 min post brushing. This pattern was observed with the no-rinse and rinse methods at 60 min, but only the no-rinse method maintained elevated fluoride levels in the sediment for up to 90 min, indicating that this effect might be more noticeable with the no-rinse method. Our findings align with previous research showing a biphasic clearance pattern, with rapid fluoride declines in the first 40–60 min followed by a slower decrease [23, 24]. The gradual decline after 60 min may result from sustained fluoride release from the sediment reservoir, suggesting it acts as a fluoride reservoir in the oral cavity that could help prevent demineralization [22, 25].

Besides the fluoride reservoir in the sediment, fluoride retention in the saliva is also influenced by other factors, including fluoride levels in the mouth, salivary flow rates, toothpaste fluoride content, rinse method, and fluoride clearance rates [16, 21, 26–29]. Salivary flow rate affects fluoride concentration in the oral cavity because a high salivary flow rate increases fluoride clearance and may reduce its concentration in the oral cavity [26]. In our study, the calculated salivary flow rate was 0.58 ± 0.20 ml/min, slightly higher than the typical unstimulated rate of 0.3–0.4 ml/min [30]. This higher rate could be a factor to consider, but our crossover design helps mitigate its impact.

We also determined a kinetic parameter, the AUC of salivary fluoride, for both post-brushing methods since AUC measures fluoride’s oral bioavailability. Our findings revealed a notably higher AUC in the no-rinse group compared to the rinse groups. A clinical study demonstrated the impact of water rinsing on salivary fluoride AUC, showing that the AUC of salivary fluoride was 2.5 times lower when water rinsing was used [11]. Therefore, our findings suggest that the no-rinse brushing method increases fluoride retention in the oral cavity and potentially lowers the risk of caries.

Absorption of high fluoride intake into the plasma is associated with the risk of dental and skeletal fluorosis. Fluoride toxicity poses serious health risks, leading to dental issues and cognitive impairment in children [13, 14, 31–33]. Dental fluorosis has been reported in individuals aged 1 to 23 years, with younger people being more susceptible [34–41]. While dental fluorosis is less common in adults, fluoride absorption can lead to skeletal fluorosis in adults [14, 40, 42, 43]. In addition, children aged 6 to 13 years have been diagnosed with this condition [44]. These conditions arise due to the absorption of high fluoride levels into the plasma.

Preventive measures and monitoring of fluoride exposure are crucial. Despite advocacy for the “spit, no-rinse” brushing method to preserve fluoride levels in the oral cavity, concerns about fluoride accumulation and the risk of fluorosis remain. Our study aims to assess fluoride concentration in plasma to validate the safety of the no-rinse method. Measuring plasma fluoride reflects systemic absorption, while urine fluoride indicates elimination. Previous studies have focused on sodium lauryl sulfate (SLS) toxicity to oral tissues [45, 46] and assumed low SLS content in toothpaste is safe with the no-rinse method [47, 48]. However, there is no evidence documenting systemic fluoride absorption with this approach. Our study aims to provide concrete data on fluoride absorption by including urine and blood evaluations for a comprehensive safety profile of the no-rinse method.

As fluoride toxicity arises from high fluoride absorption into plasma, analyzing plasma fluoride levels is an approach to assess the safety of the no-rinse method. To minimize intrusion, we collected blood samples at a single time point, 1 h after brushing, based on fluoride’s absorption half-life of 30 min [49]. This timing allowed us to estimate peak fluoride absorption. Our study observed similar mean blood fluoride levels of approximately 0.02 ppm for both the no-rinse and rinse methods. These levels are below those reported in patients with dental and skeletal fluorosis (0.16–1.25 ppm) [13, 50]. A study demonstrated that less water use for rinsing leads to higher fluoride absorption into the plasma [51], as well as The United States Environmental Protection Agency (EPA) states that a higher amount of fluoride exposure is linked to the severity of fluorosis [52, 53]. Our results showed that even though the no-rinse method leads to higher fluoride exposure, the plasma fluoride levels remained within the safety limit (0.02 ppm) [50] and were similar to those observed with the rinse method. This data provides insights into the safety of the no-rinse method after a single brushing. However, further long-term studies are needed to assess the effects of repeated use of no-rinse toothpaste formulas compared to rinsing methods.

Since urinary fluoride directly reflects fluoride excretion after absorption into the plasma, our study assesses fluoride levels in urine at various time points after brushing with either the no-rinse or rinse method. Our study found no significant difference in the amount of fluoride (µg) in urine samples between the no-rinse and rinse groups. In addition, the amount of fluoride in urine from 1 to 6 h after no-rinse brushing is comparable to the amount of baseline urine fluoride (32.54 ± 24.11 µg). This indicates minimal fluoride entered the bloodstream after brushing with the no-rinse method in this study. According to previous studies, patients with dental or skeletal fluorosis had urine fluoride levels ranging from 0.7 to 11.4 ppm, which tend to be higher in adults than in children [53, 54]. In contrast to the previous study, the urine fluoride concentrations after no-rinse brushing at all time points in our study ranged from 0.23 to 0.29 ppm, which is far below the urine levels reported from the previous studies. These findings suggest that the single using of the no-rinse method has minimal impact on overall fluoride exposure in the body.

The calculated half-life of fluoride in the no-rinse formula toothpaste was approximately 3 h and 19 min from the no-rinse method and 2 h and 54 min from the rinse method. This is similar to the recorded half-life reported previously in animal studies: fluoride half-life is generally 3–10 h [6]. The renal clearance of fluoride was measured at 14.36 ml/min and 11.85 ml/min in the no-rinse and rinse groups, respectively. These findings align closely with a previous study of renal fluoride clearance in healthy adults, which typically ranges from 12.4 to 71.4 ml/min [55]. Notably, renal clearances calculated from our study were lower than previous findings in children and adolescents which was 30–40 ml/min [56]. The differences in renal clearance observed between our study and previous research may be due to the underlying renal diseases present in the subjects of the prior study. In contrast, our research focused exclusively on healthy young adults. In addition, factors such as urinary pH, urine flow rate, and glomerular filtration rate variance between individuals can also influence the renal clearance of fluoride [56, 57]. In terms of volume of distribution, it was determined to be 4.53 L with the no-rinse method and 3.09 L with the rinse method. The low volume of fluoride distribution suggests minimal distribution into other tissues or fat compartments [58]. However, fluoride remains concentrated within the bloodstream and is cleared effectively by the kidneys. This implies that urine can reflect systemic fluoride levels. Since approximately 50% of fluoride from the bloodstream can be transferred to calcified tissues [6, 59–61], our study acknowledges the limitation of providing a partial representation of systemic absorption.

We selected the no-rinse toothpaste to use in this study for its convenience and reduced toxicity to oral tissues. Unlike conventional toothpaste, this formulation excludes sodium lauryl sulfate (SLS), paraben, sugar, plastic microbeads, and alcohol. The absence of SLS, known to be toxic to human gingival fibroblasts [46], makes it more suitable for sensitive oral tissues. SLS in toothpaste can influence fluoride pharmacokinetics by decreasing fluoride uptake to the enamel, disrupting plaque biofilm, and increasing fluoride release from oral reservoirs into saliva [62, 63]. By using the same toothpaste for both methods, we ensured that differences in fluoride absorption were due to the rinsing method alone. For future studies, including a conventional toothpaste with 1000–1500 ppm fluoride as a positive control could help validate our findings.

We acknowledge some confounding variables in our study, such as baseline oral health status and hygiene practices, which could not be fully controlled. While we screened participants for baseline oral health conditions, variability still exists. Additionally, using other dental products such as mouthwash containing fluoride during the study could influence the no-rinse method’s effects. Future research should control for these variables for more precise data.

We also recognize the limitation that our safety data is based on a single brushing with a specific no-rinse toothpaste. Further investigations are needed to evaluate the long-term use of the no-rinse method, compare different toothpaste formulations with conventional ones, and assess the safety of other ingredients in the toothpaste. Our fluoride data may not apply to all age groups, as fluoride accumulation and excretion vary with age [60]. Children’s ability to control fluoride ingestion post-brushing is also our concern, which could increase their risk of dental fluorosis with the no-rinse method. Therefore, further studies involving more diverse populations and various oral conditions, such as periodontitis, are necessary to generalize our safety results. Despite these limitations, our study provides valuable insights into optimizing fluoride toothpaste usage to maximize benefits while minimizing risks.

Overall, our study investigates the safety profile of fluoride, specifically addressing concerns about systemic absorption when brushing without rinsing. This research provides critical information for clinical recommendations and guidelines to enhance oral health benefits while minimizing the risk of fluorosis. To ensure internal validity, we randomly assigned participants into two groups and used a cross-over design, allowing the same participants to test the no-rinse and rinse methods. We standardized variables by starting the experiment 2 h after breakfast, using the same amount (1 g) and type of fluoride toothpaste, and providing low-fluoride food and drinking water to minimize fluoride intake from other sources [64, 65]. These controls strengthened our study’s internal validity. Although our findings from young adults may not apply to all age groups, however, the methodology we developed and validated for assessing fluoride toxicity can be applied to future studies involving children and other populations. Additionally, the brushing technique used in our study follows guidelines recommended by the ADA and BDA, making it relevant to various aspects of oral hygiene care. In summary, minimal fluoride entered the plasma after brushing without rinsing, indicating potential safety in a controlled experiment.

## Conclusions

1. The no-rinse tooth brushing method demonstrated higher efficacy than the rinse method by preserving fluoride concentrations above levels necessary to prevent demineralization until 30 min after a single brushing.
2. Sediment, particularly from the no-rinse method, acts as a reservoir for salivary fluoride, which effectively retains fluoride levels over an extended period.
3. Analysis of fluoride levels in plasma and urine following no-rinse and rinse methods revealed no significant difference in single exposure to fluoride toothpaste formulated for no-rinse use.
4. Within the limitation of this study, we provide information on the safety profile of fluoride after a single brushing of no-rinse formula toothpaste, particularly addressing concerns regarding potential systemic absorption when brushing without subsequent rinsing.

## Data Availability

The datasets supporting the conclusions of this article are included within the article. Further enquiries can be directed to the corresponding author.

## Abbreviations

ADA: American Dental Association
AUC: Area under the curve
BMI: Body mass index
EPA: Environmental Protection Agency
GEE: Generalized Estimating Equation
GFR: Glomerular filtration rate
k_e_: Elimination rate constant
t_1/2_: Half-life
V_d_: Volume of distribution
